# Molecular cytogenetic analysis of multi-miscarriage products of conception in clinical cases from Al-Anbar Governorate, west of Iraq

**DOI:** 10.1016/j.sjbs.2024.103932

**Published:** 2024-01-12

**Authors:** Mushtak T.S. Al-Ouqaili, Rafal M. Murshid, Basma Y. Abd Al-Kariem, Bushra A. Kanaan, Ahmed T.S. Al-Neda

**Affiliations:** a Department of Microbiology, College of Medicine, University of Anbar, Al-Anbar Governorate, Ramadi City, Iraq; b Department of Gynecology and Obstetrics, College of Medicine, University of Anbar, Al-Anbar Governorate, Ramadi City, Iraq; c Department of Obstetrics and Gynecology, Al-Ramadi Teaching Hospital for Child and Maternity, Al-Anbar Health Office, Al-Anbar Governorate, Ramadi City, Iraq; d Department of Medical Microbiology, College of Medicine, University of Anbar, Al-Anbar Governorate, Ramadi City, Iraq; e Department of Field Crops, College of Agriculture, University of Anbar, Ramadi City, Iraq

**Keywords:** Miscarriage, Fetal chromosomal abnormalities, Cytogenetic, Long-term culture

## Abstract

Most clinical miscarriages often occur throughout the first trimester of pregnancy, with fetal chromosomal abnormalities being identified as the primary reason for such occurrences. The objective is to analyze the fetal chromosomal aberrations in the product of conception among Iraqi patients suffering from recurrent miscarriages. The cross-sectional study was performed on 60 cases of products of conception in women suffering from multiple miscarriages, obtained from Department of Obstetrics and Gynecology is located in Ramadi Teaching Hospital for Child and Maternity, as well as other Private Clinics in the Ramadi City. Long-term culture of conventional cytogenetic analysis using the G-banding technique was employed to determine the chromosomal disorder of fetal tissue part or villus samples. Fetal chromosomal abnormalities were detected in 86.7 %. Numerical chromosomal abnormalities were revealed in 98.1 %, while structural abnormalities were detected in 1.9 %. Additionally, the commonest gestation loss occurs in parents under 35 years in the first trimester (92.3 %). Trisomy 21 was the most frequent (46.2 %) in gestational loss. Fetal chromosomal abnormalities have been linked with gestational loss in Iraqi couples. Therefore, it is recommended that cytogenetic analysis should be performed to identify the genetic cause of recurrent miscarriage. This is important for providing appropriate genetic counseling and educating couples about the risk of future pregnancies.

## 1 Introduction

Recurrent miscarriages have been recognized as implantation failures in natural conception. These conditions, commonly known as recurrent pregnancy losses or habitual abortions (Khamees and Al-Ouqaili, 2022). It is also defined as a spontaneous termination of a clinically confirmed intrauterine pregnancy before the fetus reaches viability, often leading to considerable emotional distress for couples seeking to conceive (Von Woon et al., 2022). Most clinical miscarriages often occur throughout the first trimester of pregnancy, with fetal chromosomal abnormalities being identified as the primary reason for such occurrences (Ozawa et al., 2019). The commonest chromosomal variations in such duration include numerical chromosomal abnormalities such as monosomy X and trisomy 21 and structural ones, which result in acquiring or losing inherited material (D’Ippolito et al., 2023). A limited number of these chromosomal imbalances may cause disturbances in a later trimester. Even chromosomal defects that can potentially be viable, such as monosomy X and trisomy 21, sometimes undergo spontaneous loss during the first twelve weeks after conception (Essers et al., 2023).

Chromosomal structural abnormalities in parents are the primary genetic cause of recurrent miscarriages. However, these abnormalities occur in only 2.78 %–4.1 % of affected couples (Tunç et al., 2016, Fan et al., 2016). In addition to genetic characteristics that may be passed down, other maternal factors have been identified as being associated with spontaneous abortion or fetal chromosomal abnormalities. These factors include maternal age, reproductive history, and immunological or endocrine dysfunction (Pinar et al., 2018). Furthermore, it has been shown that an increased homocysteine concentration in the maternal blood is associated with a heightened likelihood of fetal loss and stillbirth. There is an established correlation between gene polymorphisms involved in folate metabolism and the occurrence of chromosome breakage and fetal chromosomal aneuploidy (Xu et al., 2022).

There is a positive correlation between paternal age and the tendency for a rise in sperm DNA fragmentation index. There was a significant correlation between elevated levels of sperm DNA damage and an increased incidence of miscarriage among patients (Haddock et al., 2021). Environmental influences are among the non-hereditary factors implicated in fetal chromosomal abnormalities. Repeated miscarriages are often associated with environmental variables, including using substances such as smoking, caffeine, and alcohol and exposure to toxic or pesticide material (Sambo and Goldman, 2023).

The cytogenetic analysis of products of conception (POC) can provide useful insights into the underlying causes of miscarriage and determine the likelihood of future recurrence for the affected couple. Nonetheless, the validity of this work hinges upon the proficient cultivation of fetal tissue and the accurate generation of metaphase cells (Pylyp et al., 2018, Holt et al., 2022).

There is limited works on the frequency of embryonic chromosomal abnormalities in Iraq and neighboring countries with recurrent miscarriages. In light of this, the objective of this study was to analyze the embryonic chromosomal aberrations in product of conception among Iraqi patients suffering from recurrent miscarriage. Further, to detect the type of such fetal abnormalities either numerical or structural and their relation to maternal and gestational ages.

## 2 Patients and methods

This study was conducted from December 2021 to September 2023 on the sixty POC (fetal tissue part or villus samples) of women suffering from recurrent miscarriage who were included in this study. All POCs were submitted to Ramadi Teaching Hospital for Child and Maternity and other private clinics in Ramadi City in western Iraq. Inclusion criteria of women include in this study were negative result of lupus anticoagulants, anti-cardiolipin antibodies, anti-phospholipid antibodies and TORCH (Toxoplasma, Rubella, Cytomegalovirus, Herpes simplex virus type1 and 2).

The product of conception samples was placed in a sterile container containing sterile saline for specimen transportation. POCs often contain large numbers of bacteria. Therefore, it must be refrigerated until subjected to the next day procedure. The transportation to the genetics laboratory has been done either by a cold pack or at room temperature. The gynecologists record of reproductive history, which included information on paternal and gestational ages.

The specimens were rejected if they had been frozen or fixed by formalin, formaldehyde, and alcohol due to being unable to be cultured, as freezing and fixation kill the cells. One challenge that can hinder the success of POC cell culture is the duration between the loss of gestation and the collection of the material. If this period is lengthy, the cells within the tissue may stop dividing, making cytogenetic analysis impossible. Maternal (deciduous) tissue cells may grow instead of fetal tissue, leading to an incorrect karyotype.

### 2.1 Ethics statement

The study obtained approval from the Medical Ethics Committee of the University of Anbar by the Helsinki Declaration. Informed consent was obtained from all subjects involved in the study.

### 2.2 Chromosomal part of the study (karyotyping)

According to Rooney (2001) protocol, the tissues underwent a conventional cytogenetic analysis involving long-term culture. The G-banding approach was utilized during this analysis. Every sample was promptly placed in a sterile cup with normal saline and transported to the medical laboratory. The specimens were dissected from endometrial tissue and blood clots by washed with fresh RPMI 1640 Medium solution (HiMedia, Mumbai, India). AmnioMAX™ complete media (Gibco Invitrogen, USA) in flasks (T-75) of cell culture were used. Fetal tissues were incubation for 7–14 days at a temperature of 37 °C and a CO2 concentration of 5 %. The metaphase cells were subsequently arrested with Colcemid (Biowest, European) and treated with a hypotonic solution (KCL 0.075) and a fixative consisting of methanol-acetic acid in a ratio of 3:1. The resulting cells were then dispersed onto microscope slides and subjected to G-banding. G-banding was performed by Geimsa and Trypsin treatment; according to standard method by Polipalli et al. (2016).

#### 2.2.1 Karyotype analysis

The chromosomal status was determined using the MetaClass Karyotyping system manufactured by Microptics S.L. in Barcelona, Spain, in conjunction with a fluorescence microscope from Euromex in Arnhem, Netherlands. The cytogenetic analysis involved the utilization of G-banding. Each individual underwent an examination of twenty metaphases. Karyotypes were recorded according to the recommendations of International System of Human Cytogenetic Nomenclature (ISCN), 2020 (Kanaan et al., 2022, Kanaan et al., 2023).

### 2.3 Statistical analysis

The statistical analysis was conducted using version 22 of the SPSS software (Armonk, NY, USA).

## 3 Results

### 3.1 Fetal chromosomal abnormalities

**Fig. 1:**
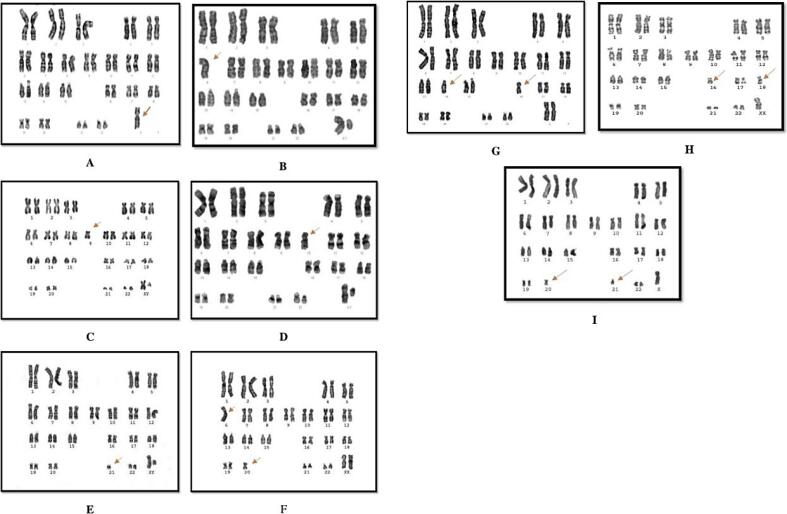
Karyotypes of embryonic chromosomal abnormalities are represented as the following: A. Monosomy X, B. Monosomy 6, C. Monosomy 9. D. Monosomy 10, E. Monosomy 21, F. Monosomy 6 and 20, G. Monosomy 14 and 16. H. Monosomy X, 16, 18 and I. Monosomy X, 20, 21.

**Fig. 2:**
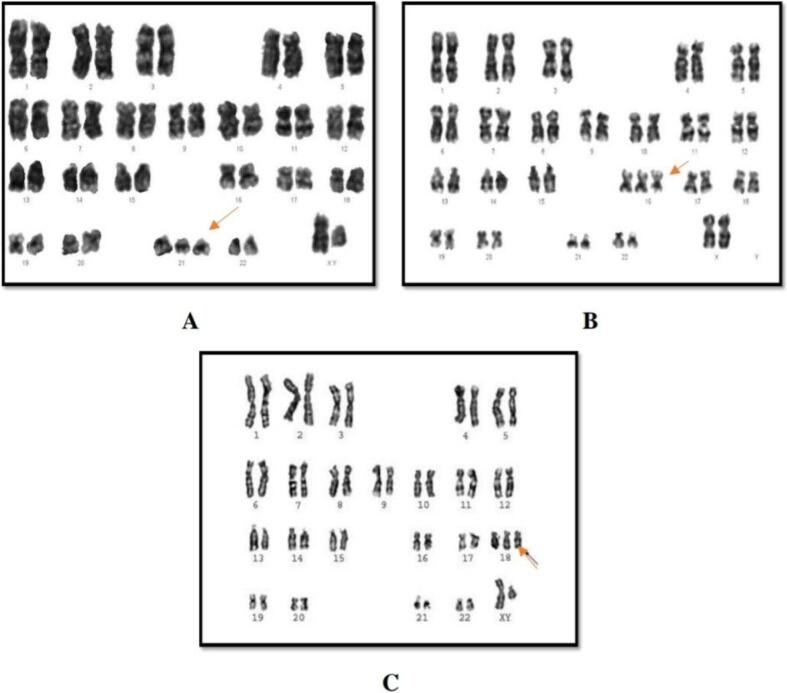
Karyotypes of embryonic chromosomal abnormalities are show as the following: A. Trisomy 21, B. Trisomy 16, C. Trisomy 18.

**Fig. 3:**
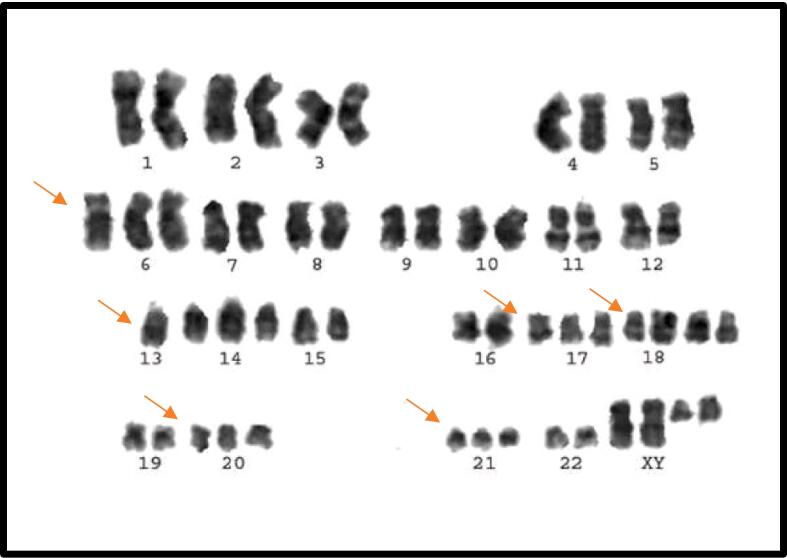
Karyotype of monosomy 13, trisomy 6, 17, 20 and 21, tetrasomy 18.

**Fig. 4:**
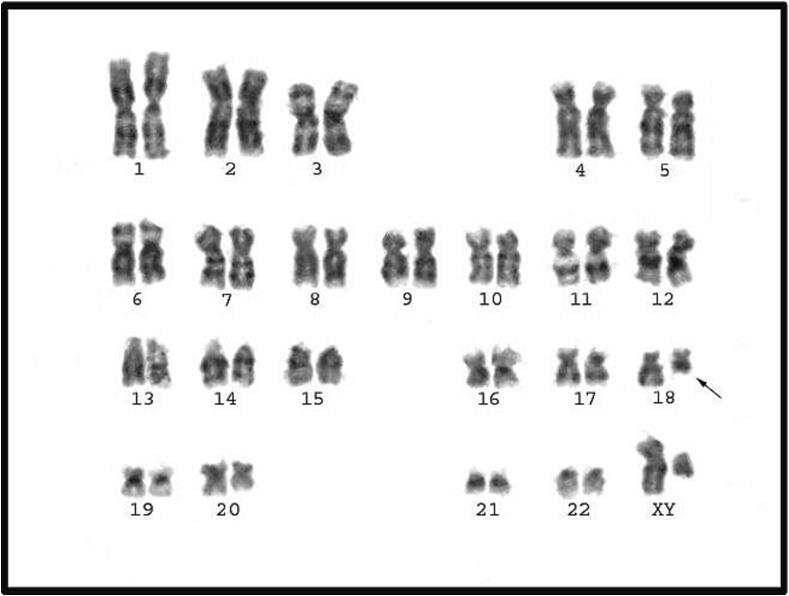
Karyotype of 46, XY, del 18 (q21- qter).

Out of 60 clinical specimens of POC, the karyotyping analysis revealed in 52 (86.7 %) with embryonic chromosomal abnormalities, while eight (13.3 %) of them had normal karyotypes. The numerical chromosomal abnormalities were detected in 51 (98.1 %) cases while One (1.9 %) with structural abnormalities. All details are represented in (Table1) and (Fig. 1, Fig. 2, Fig. 3, Fig. 4).

**Table 1:** Cytogenetic variation, karyotype, and frequency of products of conception.

Cytogenetic variation	Karyotype	No. of case	Frequency (%)
Normal karyotype	46, XX (2) 46, XY (3)	8	13.3 %
Abnormal Karyotype		52	86.7 %
A-Aneuploidy		51	98.1 %
Monosomy X	45, X	14	26.92 %
Monosomy 6	45, XY, −6	1	1.92 %
Monosomy 9	45, XY, −9	1	1.92 %
Monosomy 10	45, XY, −10	1	1.92 %
Monosomy 21	45, XY, −21	1	1.92 %
Monosomy 6 and 20	44, XY, −6, −20 44, XX, −6, −20	2	3.85 %
Monosomy 14 and 16	44, XX, −14, −16	1	1.92 %
Monosomy X, 16, 18	43, X, −16, −18	1	1.92 %
Monosomy X, 20, 21	43, X, −20, −21	2	3.85 %
Trisomy 21	47, XX, +21 47, XY, +21	24	46.2 %
Trisomy 16	47, XX, +16	1	1.92 %
Trisomy 18	47, XY, +18	1	1,92 %
Monosomy 13, trisomy 6, 17, 20 and 21, tetrasomy 18	54, XXYY, +6, −13, +17, +18, +20, +21	1	
B-Structural abnormalities		1	1.9 %
Deletion in 18	46, XY, del 18 (q21 qter)	1	1.9 %

#### 3.1.1 Aneuploidy chromosome karyotype

**Hypoploidy:**

The karyotype of autosomal monosomy of embryonic chromosomal abnormalities were detected and distributed into the following: Two (3.85 %) with monosomy 6 and 20, One abnormal case (1.92 %) was detected for each monosomy 9, monosomy 10, monosomy 21, monosomy 14 and 16, monosomy X, 16 and 18, monosomy X, 20 and 21. Pure monosomy X karyotype (45, X) was detected in 14 (26.92 %) cases of the product of conception (Fig. 1).

**Hyperploidy:**

The karyotypes of numerical chromosomal abnormalities were distributed into the following: - Trisomy 21 in 24 (46.2 %) followed by one abnormal case (1.92 %) detected for each of the trisomy 16 and trisomy 18 (Fig. 2).

**Multiple chromosome abnormalities:**

In cases (1.92 %), POC was detected with not one chromosomal alteration. Karyotype was hypoploidy at monosomy 13, Hyperploidy at trisomy 6, 17, 20, 21, and tetrasomy 18 (Fig. 3).

#### 3.1.2 Structural chromosomal abnormalities

The distinguished and only One case (1.9 %) of structural chromosomal abnormalities yielded in chromosome 18 (Fig. 4).

### 3.2 Maternal age with fetal chromosomal abnormalities

In 60 cases, maternal age ranged from 19 to 46 years. Patients were categorized as young mothers (<35 years; n = 49) and advanced mothers (≥35 years; n = 11). This study revealed more embryonic chromosomal abnormalities of POC in young mothers than in advanced maternal age. Out of 52 cases of chromosomal abnormalities, 41 (78.8 %) cases were detected in young maternal age, while 11 (21.2 %) cases were in advanced maternal age (Table 2).

**Table 2:** Role of maternal age with chromosomal abnormalities of product of conception.

Karyotype variation	Maternal age	
	<35 years	>35 years
Monosomy X	12 (29.26 %)	2 (18.2 %)
Monosomy 6	1 (2.44 %)	0 (0.0 %)
Monosomy 9	1(2.44 %)	0 (0.0 %)
Monosomy 10	1 (2.44 %)	0 (0.0 %)
Monosomy 21	1 (2.44 %)	0 (0.0 %)
Monosomy 6 and 20	1 (2.44 %)	1 (9.1 %)
Monosomy 14 and 16	1 (2.44 %)	0 (0.0 %)
Monosomy X, 16, 18	1 (2.44 %)	0 (0.0 %)
Monosomy X, 20, 21	2 (4.88 %)	0 (0.0 %)
Trisomy 21	18 (43.9 %)	6 (54.5 %)
Trisomy 16	1 (2.44 %)	0 (0.0 %)
Trisomy 18	0 (0.0 %)	1 (9.1 %)
Multiple-chromosomal abnormalities	0 (0.0 %)	1 (9.1 %)
Structural abnormalities	1 (2.44 %)	0 (0.0 %)
Total	**41 (78.8 %)**	**11 (21.2 %)**

In young maternal age were detected 18 (43.9 %) cases of trisomy 21, 12 (29.26 %) cases of monosomy X, and two (4.88 %) cases of monosomy X, 20, and 21. One (2.44 %) case of embryonic chromosomal abnormalities was detected for each of the following: Monosomy 6, monosomy 9, monosomy 10, monosomy 21, monosomy 6 and 20, monosomy 14 and 16, monosomy X, 16, 18, trisomy 16. Structural abnormalities were detected in one (2.44 %) case.

In advanced maternal age were detected numerical chromosomal abnormalities in six (54.5 %) cases of trisomy 21, two (18.2 %) cases of monosomy X, one (9.1 %) case of trisomy 18, one (9.1 %) case of monosomy 6 and 20. Multiple chromosomal abnormalities were detected in one (9.1 %) case.

### 3.3 Fetal chromosome abnormalities and age of gestation

The 60 cases where the gestational ages were available were determined using the ultrasound scan. According to the distribution of the gestational age, the study result revealed that in most of the study cases, 50 (83.3 %) occurred in the first trimester, followed by seven (11.7 %) in the second trimester and three (5.0 %) in the third trimester. Moreover, embryonic chromosomal abnormalities were detected in 44 (88.0 %) cases in the first trimester, while normal karyotype was six (12.0 %) cases. Six (85.7 %) cases in the second trimester had chromosomal abnormalities, while one (15.3 %) had a normal karyotype. Chromosomal abnormalities and normal karyotype were detected in two (66.6 %) cases and one case (33.3 %) in the third trimester, respectively.

Further, the commonest chromosomal aberrations were trisomies 21 (42.0 %), followed by monosomies X in 13 cases (26 %) and monosomy X 20, 21 in 2 cases (4.0 %), as represented in Table 3.

**Table 3:** Distribution of chromosome abnormalities according to gestational age.

Cytogenetic variation	Age of gestation		
	First trimester	Second trimester	Third trimester
Normal	6 (12.0 %)	1 (14.28 %)	1 (33.3 %)
Monosomy X	13 (26.0 %)	1 (14.28 %)	0 (0.0 %)
Monosomy 6	0 (0.0 %)	0 (0.0 %)	1 (33.3 %)
Monosomy 9	1 (2.0 %)	0 (0.0 %)	0(0.0 %)
Monosomy 10	1 (2.0 %)	0 (0.0 %)	0(0.0 %)
Monosomy 21	1 (2.0 %)	0 (0.0 %)	0 (0.0 %)
Monosomy 6 and 20	0 (0.0 %)	2 (28.57 %)	0 (0.0 %)
Monosomy 14 and 16	1 (2.0 %)	0(0.0 %)	0 (0.0 %)
Monosomy X, 16, 18	0 (0.0 %)	1 (14.28 %)	0 (0.0 %)
Monosomy X, 20, 21	2 (4.0 %)	0 (0.0 %)	0(0.0 %)
Trisomy 21	21 (42.0 %)	2 (28.57 %)	1 (33.3 %)
Trisomy 16	1 (2.0 %)	0 (0.0 %)	0 (0.0 %)
Trisomy 18	1 (2.0 %)	0 (0.0 %)	0 (0.0 %)
Multiple-chromosomal abnormalities	1 (2.0 %)	0 (0.0 %)	0 (0.0 %)
Structural abnormalities	1 (2.0 %)	0 (0.0 %)	0 (0.0 %)
Total	**50 (83.3 %)**	**7 (11.7)**	**3 (5.0 %)**

## 4 Discussion

Recurrent miscarriage is a widely studied phenomenon characterized by multiple contributing elements, encompassing parental genetic abnormalities, uterine irregularities, immunological illnesses, hematological conditions, hormone imbalances, and environmental influences. During the first trimester, the main reason for spontaneous miscarriage is often because of chromosomal abnormalities, which can include chromosomal instability, single gene mutations, and abnormalities in the sperm's chromosomal composition. These factors help to explain the underlying causes of unexplained reproductive loss (Hardy and Hardy, 2018).

Several molecular techniques have been used for the genetic study of POC. However, the emphasis has primarily been on cytogenetic findings for the following explanations: Firstly, in clinical practice, many women choose conventional karyotyping rather than molecular testing due to its lower cost. Secondly, it is important to note that chromosomal abnormalities have a detrimental impact and are associated with miscarriage. On the other hand, submicroscopic aberrations may be viable and may have a role in the development of neurodevelopmental problems or prenatal ultrasonography abnormalities (Wu et al., 2021).

Genetic testing results for POCs provide an important information for reproductive counselling. This includes elucidating the cause of reproductive loss in instances where chromosomal abnormalities are identified and prompting further investigation into alternative causes in cases where the karyotype appears normal. In addition, it is important to note that imbalanced forms of family chromosomal abnormalities may be detected, hence necessitating the performance of cytogenetic analysis on a pair (Pylyp et al., 2018).

In this study, the cytogenetic analysis of POC revealed that embryonic chromosomal abnormalities were detected in 86.7 % while the normal karyotype appeared in 13.3 %. Additionally, the commonest loss of gestation occurs in young maternal age (<35 years) in the first trimester period of pregnancy (83.3 %). Embryonic chromosomal abnormalities were revealed in the first trimester 44 (84.6 %), followed by Six cases (11.5 %) and Two cases (3.9 %) of the second and third trimester respectively. Based on previous studies, a significant proportion of miscarriages occurring in the first trimester of pregnancy can be caused by non-hereditary chromosomal aneuploidy. This observation suggests the potential influence of environmental variables on the development of abnormal chromosomal structures in embryos, leading to spontaneous abortion (Larsen et al., 2013).

It is hypothesized that a potential association was observed between increased susceptibility to stillbirth and spontaneous abortion and exposure to air pollutants, specifically particulate matter (PM), cooking smoke and carbon monoxide (CO). Exposure to PM10 (particles with a diameter of 10 µm or less, these particles are small enough to pass through the throat and nose and enter the lungs) for the entirety of pregnancy is correlated with an elevated likelihood of experiencing spontaneous abortion. Exposure to CO through the first trimester of pregnancy was linked with a heightened likelihood of spontaneous miscarriage. Furthermore, the exposure to CO during the last trimester of pregnancy was connected with an elevated danger of stillbirth. Moreover, it has been observed that the emission of cooking smoke is associated with an elevated likelihood of stillbirths, and this correlation has been consistently supported by available evidence (Grippo et al., 2018). Yang et al. (2018) discovered a lack of adequate and contradictory information about several other contaminants, including NO2 and SO2. In contrast, Haddock et al. (2021) conducted research that demonstrated additional male characteristics, including occupation, environmental exposure, and smoking, which have been found to impact sperm quality and thus contribute to early embryo loss. The generation of reactive oxygen species (ROS), such as through heightened stress levels, participation in competitive sports, exposure to infections, consumption of alcohol, smoking, nicotine, and substance misuse, has the potential to lead to the fragmentation of sperm DNA (Al-Qaisi et al., 2020, Al-Ouqaili et al., 2022).

A previous study by Soler et al. (2017) in Barcelona (Spain), reported that fetal chromosomal abnormalities detected in the first trimester of gestational age were 70.3 %. In contrast, Pylyp et al. (2018) found that in a study of Ukrainian POCs, 50.1 % of first-trimester miscarriages had chromosomal abnormalities. In a China study by Zhang et al. (2021), 165 (48.53 %) were identified with fetal chromosomal abnormalities. The above studies' results agreed with those observed in our study.

In the current study, out of 52 (86.7 %) cases of chromosomal abnormalities, numerical chromosomal abnormalities were 51 (98.1 %) cases, while structural abnormalities were one case (1.9 %). In a previous study conducted by Wu et al. (2021), it was determined that 667 (53.2 %) chromosomal abnormalities had been discovered. Among these aberrations were 592 (47.3 %) numerical abnormalities, 38 (3.0 %) structural abnormalities, and 37 (3.0 %) mosaic aberrations cases.

In the study results, it has been observed that monosomy X represents the second most prevalent chromosomal abnormality after trisomy 21. This discovery proves the hypothesis that the fatality observed in individuals with 45, X chromosomal abnormalities can be attributed to the absence of “rescue” cell lines, namely those with a 46, XX karyotype, in crucial tissues necessary for proper development, such as the placenta (Hook and Warburton, 2014).

The previous study investigating the correlation between polycystic ovary syndrome (PCOS) and recurrent miscarriage revealed that PCOS is associated with a roughly threefold increase in the probability of experiencing miscarriages. On the other hand, another investigation did not establish a significant relationship between the two variables. Among these, it is observed that miscarriages in individuals with PCOS predominantly occur during the initial trimester of pregnancy. Nevertheless, the impact of PCOS on pregnancy might exhibit significant variability between different individuals. Certain individuals with PCOS experience favourable outcomes regarding healthy pregnancies, while others may encounter miscarriages or additional problems (Boomsma et al., 2008, Yu et al., 2016).

The study found that fetal chromosomal abnormalities were detected in 86.7 % of cases. Of these, numerical chromosomal abnormalities were found in 98.1 % of cases, while structural abnormalities were identified in only 1.9 %. The most common cause of gestational loss was found to be in materials under the age of 35 in the first trimester, accounting for 92.3 % of cases. Trisomy 21 was the most frequent chromosomal abnormality, accounting for 46.2 % of cases. The study also found that there were a high number of embryonic chromosomal abnormalities among Iraqi couples experiencing gestational loss. As a result, it is recommended that cytogenetic analysis must be performed to determine the genetic basis of recurrent miscarriage. The result of karyotyping will help in the presentation of a genetic counselling for educating couples about the risks associated with future pregnancies.

## Declaration of competing interest

The authors declare that they have no known competing financial interests or personal relationships that could have appeared to influence the work reported in this paper.
